# COVID-19 and primary care in six countries

**DOI:** 10.3399/bjgpopen20X101128

**Published:** 2020-09-09

**Authors:** Patricia Huston, John Campbell, Grant Russell, Felicity Goodyear-Smith, Robert L Phillips, Chris van Weel, William Hogg

**Affiliations:** 1 University of Ottawa, Faculty of Medicine, Department of Family Medicine, Ottawa, Canada; 2 University of Ottawa, Faculty of Medicine, School of Epidemiology and Public Health, Ottawa, Canada; 3 University of Exeter Medical School, University of Exeter, Exeter, UK; 4 Monash University, Department of General Practice, Clayton, Australia; 5 University of Auckland, Faculty of Medical & Health Sciences, Department of General Practice & Primary Health Care, Auckland, New Zealand; 6 Center for Professionalism and Value in Health Care, American Board of Family Medicine, Washington, DC, US; 7 Radboud University Medical Center, Department of Primary and Community Care, Nijmegen, The Netherlands; 8 Australian National University, Department of Health Systems Research and Policy, Canberra, Australia; 9 Institut du Savoir, Hôpital Montfort, Ottawa, Canada

**Keywords:** COVID-19, Public health, Primary care, Physicians, family, General practitioners, Communicable diseases

## Introduction

With the focus of the COVID-19 pandemic on the many challenges in public health, acute and long-term care, what has happened within primary care has remained largely below the radar. Yet primary care physicians (family doctors and GPs) can constitute up to 50% of the medical workforce^1^ and are highly susceptible to contracting emerging infectious diseases themselves, as they are often the first point of contact people have with the health system.^2^ This article comments on what is happening to primary care provision in six well-resourced countries: Australia, New Zealand (NZ), Canada, the Netherlands, the UK, and the US. Although primary care has been on the front line with COVID-19 cases, this has come at great cost.

In all six countries, primary care physicians participated in the initial assessment and triage of people with possible COVID-19, although how that was done varied between countries, and was rarely in the physician’s office (Table 1). They decided who could be managed at home, and who needed specialist referral or hospital admission. Physicians often deferred routine follow-up visits in their offices, instead offering patients remote assessments by telephone, email, and videoconferencing, and assisting in assessment centres.

**Table 1.** Site of primary care assessment of potential COVID patients in six countries.^a^

**Country**	**Virtual** ^b^(**off-site**)	**Primary care offices**	**Assessment centres**	**After-hours clinics**	**Emergency** **departments**	**Home visits**
Australia	Common	Occasional	Common^c^	Sometimes	Common	Occasional
New Zealand	Common	Sometimes	Common	Sometimes	Occasional	Sometimes
Canada	Common	Occasional	Common	Sometimes	Common	Occasional
The Netherlands	Common	Common	Occasional	Common^d^	Common	Sometimes^e^
UK	Common	Occasional	Common	Sometimes^d^	Sometimes	Occasional
US	Common	Sometimes	Common	Sometimes	Sometimes	Occasional

^a^This is based on knowledge of national pandemic plans, professional communications and anecdotal evidence amassed by the authors in order to give a general picture; it does not reflect the heterogeneity that may occur within each country. ^b^Virtual assessments were conducted to advise patients as to whether it was necessary to be clinically assessed and, if indicated, to direct them to the appropriate venue. ^c^Known as GP respiratory clinics. ^d^Seeing patients in off-hours is part of primary care system. ^e^Commonly done for the sub-population of vulnerable older persons.

Countries differed, however, in terms of pre-existing universal health coverage, pandemic readiness, and the level of government and public support for public health measures. We define support for public health measures as endorsement and general abidance with travel restrictions, physical distancing, use of masks, and minimising numbers of social contacts.

Of the six countries, Australia and NZ were the best prepared. Both had universal health coverage and up-to-date pandemic plans. In Australia, guidelines were in place for GPs for both pandemic influenza and public health emergencies.^3,4^ Widespread testing was rapidly established, and national and state governments assisted in the provision of personal protective equipment (PPE) to general practice.^5^ NZ also had an updated pandemic plan, and District Health Boards led the pandemic response with generally good coordination of primary care and public health.^6^ Some government support was provided to primary care for their increased workload, although many practices still had a shortfall.^7^ Until July 2020, both Australia and NZ had relatively few cases of COVID-19^8^ (Table 2). This is believed to be due to early implementation of travel restrictions, comprehensive physical distancing, intense community-based testing, and strong government and public support for the health measures. Currently most cases in NZ are in returning New Zealanders, picked up during mandatory quarantine. However, at the time of submission, Melbourne, Australia began experiencing a substantial second wave of active infections. An extensive public health response has been required to deal with the problem, the cause of which appears linked with major deficiencies in the management of international travellers in quarantine.

**Table 2.** COVID-19 cases, deaths, case-fatalities, and mortality per 100 000 people in six well-resourced countries

**Country**	**Confirmed cases**	**Deaths**	**Case-fatality**	**Death per 100 000 population**
UK	321 064	41 454	12.9%	62.35
US	5 438 325	170 497	3.1%	52.11
The Netherlands	64 980	6194	9.5%	35.95
Canada	124 218	9075	7.3%	24.49
Australia	23 773	438	1.8%	1.75
New Zealand	1643	22	1.3%	0.45

Confirmed cases and hence case-fatality depend on the degree to which community testing and surveillance is occurring, and whether all deaths (including those at home or in aged care facilities) are included.

As of 18 Aug 20. Source Johns Hopkins Coronavirus Resource Center^8^ https://coronavirus.jhu.edu/data/mortality.

Canada, the Netherlands, and the UK all have universal health coverage, but had varying degrees of pandemic readiness. Canada had a recently updated pandemic plan and identified primary care as responsible for assessing ambulatory patients.^9^ It had a moderate number of cases,^8^ due to initial delays in testing and in implementing travel restrictions, challenges encountered in maintaining PPE supplies, and in sub-optimal infection control in long-term care facilities. However, there has been strong government and public support for the public health measures.

The Netherlands has universal health coverage and had a current pandemic plan. It incentivised family physicians to assess all potential COVID-19 patients.^10,11^ Within the first week of the epidemic, family practices were able to reorganise their flow of patients from mainly face-to-face to virtual consultations, and separate practice visits of suspected from non-COVID patients.^1^ However, initial limited access to testing and PPE resulted in a large number of nursing home residents and healthcare professionals becoming infected.^12^ With strong government and public support for the public health measures, the Netherlands has had a moderate number of cases (Table 2).^8^

The UK has universal health coverage but their pandemic influenza strategy was published in 2011, with latest updates in 2014.^13^ Although this strategy only mentioned primary care once, primary care responded quickly, with GP practices reorganising access and facility arrangements to adapt to the emerging demands. The UK has had a large number of cases (Table 2),^8^ thought to be due to the initial lack of widespread testing and PPE, changing responses creating public uncertainty, and substantial regional variation which suggests sociodemographic factors as drivers of infection.

The US does not have universal health coverage. Although the US pandemic plan had been updated in 2017, primary care had no defined role.^14^ The National Security Council Directorate for Global Health Security and Biodefense, set up to manage US response to disease outbreaks and pandemics, was disbanded in 2018. Primary care was largely left on its own to assist in the response, and to deal with changing, confusing, and at times conflicting recommendations from local, state, medical specialty, and federal authorities.^15^ Primary care physicians had to find their own PPE for office visits and most were dependent on fee-for-service funding. Prior to COVID-19 telehealth was uncommon, so the rapid pivot to virtual visits happened without consistent changes in payment support and required a leap of faith by physicians that they would remain solvent. The US has had a quarter of the world’s cases of COVID-19 (Table 2),^8^ thought to be due to delays in testing, inconsistent messaging, variability in social distancing guidelines, health disparities accentuated by decreased access to health care, and variable support for public health guidelines by governments and the public.^16,17^

## Decrease in use of primary care services in some jurisdictions

Although primary care physicians rose to the challenge of the pandemic, this has come at a cost. In all countries, emergency plans did not clearly identify how primary care could be sustained in a prolonged pandemic scenario.

There has been documented decreases in the use of primary care services in the US, Canada, and the Netherlands.^12,18,19^ This is thought to be largely because patients have not sought care due to an impression that the healthcare system was already overstretched, a perceived increased risk of COVID-19 exposure in healthcare settings, and directives to self-isolate at home.

In all six countries, there are now growing concerns regarding the increased risk of morbidity and mortality from deferred referrals and routine primary care.^20–24^ It has been estimated that as many as 75 000 Americans could die from drug or alcohol misuse and suicide linked to the coronavirus pandemic.^25^ Cardiologists have reported marked decreases in people presenting with myocardial infarctions both in the US^26^ and Europe.^27^ There is a documented decline in childhood immunisation coverage in the UK and the US, which increases the risk of new outbreaks from vaccine-preventable diseases.^28,29^

## Loss of primary care capacity in some countries

The primary care physicians who have been the most adversely affected by COVID-19 are those with independent businesses who are paid on a fee-for-service basis. This is the most common form of remuneration in the US and Canada, and is a large part of the remuneration in Australia and NZ. With the rapid decrease in patient visits, either no or low fees for telehealth consultations, and delayed remuneration, some US and Canadian physicians have faced severe financial losses. Some have had to furlough staff, a number have closed their offices, and some are filing for bankruptcy.^18,19,30–33^ Although physicians have had some economic challenges in the other four countries, this has been mitigated by additional payments made for COVID care, better remuneration for telehealth, and blended payment models where fee-for-service is only part of their remuneration package. The best situation is in NZ, where for 100 days there was no physical distancing measures, practices were seeing patients face-to-face without restriction, and patient volumes were high. However, at the time of publication, Auckland residents were back in lockdown due to the emergence of a community-based cluster.

## Discussion

COVID-19 is exposing health system fragility, not only in the public health, acute and long-term care systems, but also in primary care. Its impact has varied from country to country but, overall, the countries that have fared the best are the ones with universal health coverage, updated pandemic plans that include primary care, and good government and public support for the public health measures. In all countries, primary care physicians have been on the front line of the pandemic response, and non-COVID-19 primary care services have decreased. Not only are there signs of increased non-COVID-19 mortality but, in countries that rely on a fee-for-service payment model, there have also been closures of primary care offices and a loss of primary care capacity. In all countries, core components of primary care have been challenged in the effort to fight COVID-19. Lockdown drastically reduced access, to some degree mitigated by telehealth. Comprehensiveness and continuity of services have suffered, as addressing the pandemic was prioritised over dealing with non-COVID-19 conditions. For those in continued lockdown, it has been difficult to provide person-centred care where patients struggle with the technology, and have increasing mental health issues. Inter-sectorial coordination of primary care with public health, secondary care, and community-based services has been key in mounting an effective pandemic response.

In all countries there is a need to carefully consider how best to sustain primary care services, including consistent public messaging regarding when it is appropriate to see a doctor. The closure of primary care offices in the US and Canada is concerning, and strategies are needed on how to strengthen primary care to withstand future health emergencies.

The aftermath of pandemics provides an opportunity to build more resilient health systems.^34^ The World Health Organization has long advocated ’health for all’.^35^ The recent Astana Declaration identifies the goal of universal health coverage.^36^ During a pandemic, the need for this becomes very clear. The Declaration also sets forth the vision of sustainable primary care within a strong health system, where all key stakeholders are aligned in supporting national health policies, strategies, and plans.^36^ The importance of this alignment is indicated by the association of better COVID-19 outcomes in countries that have had good government and community support for public health measures.

Why do we need sustainable primary care for a strong health system response to pandemics? Primary care is where most health care takes place, and where most people have trusted health-related relationships; its physicians are the ’eyes and ears’ of the health system. As influenza surveillance has demonstrated, primary care can provide important data to public health.^37–43^ This can be expanded with the use of patient surveys that link with electronic medical records to collect and combine real-time data on emerging symptoms, complications, patient responses to public health messaging, adaptive coping mechanisms, and more. However, this can only be done if primary care has adequate resourcing and can strengthen its capacity to maintain essential services.

COVID-19 is testing the resilience of health systems, even in well-resourced countries. Its challenges have brought into focus the need to protect our global health with more sustainable primary care within a well-coordinated health system that has strong government and public support for its policies.

## References

[1] Canadian Institute of Health Information (2018). Physicians in Canada, 2018: summary report. https://www.cihi.ca/sites/default/files/document/physicians-in-canada-2018.pdf.

[2] Gamio L (15 Mar 2020). The Workers Who Face the Greatest Coronavirus Risk. The New York Times [online]. https://www.nytimes.com/interactive/2020/03/15/business/economy/coronavirus-worker-risk.html.

[3] Royal Australian College of General Practitioners (2017). Managing emergencies in general practice: a guide for preparation, response and recovery. Updated June 2017. https://www.racgp.org.au/download/Documents/e-health/Managing-emergencies-in-general-practice.pdf.

[4] Royal Australian College of General Practitioners (2014). Managing pandemic influenza in general practice: a guide for preparation, response and recovery. https://www.racgp.org.au/download/Documents/Guidelines/Flukit/pandemic-flu-kit.pdf.

[5] Tsirtsakis A (8 Apr 2020). PHNs to receive 2.3 million masks to help fight coronavirus. News GP [online]. https://www1.racgp.org.au/newsgp/professional/primary-health-networks-to-receive-2-3-million-mas.

[6] Ministry of Health (2017). New Zealand Influenza Pandemic Plan: A framework for action 2017 (2nd edn). Wellington: Ministry of Health. https://www.health.govt.nz/publication/new-zealand-influenza-pandemic-plan-framework-action#:~:text=The%20New%20Zealand%20Influenza%20Pandemic,respond%20to%20an%20influenza%20pandemic.&text=It%20also%20provides%20general%20information,for%20the%20New%20Zealand%20public.

[7] TAS (2020). Primary Health Organisation Service Agreement Amendment Protocol (PSAAP). https://tas.health.nz/dhb-programmes-and-contracts/primary-care-integration-programme/primary-health-organisation-service-agreement-amendment-protocol/.

[8] Worldometer (2020). Coronavirus / countries. https://www.worldometers.info/coronavirus/countries-where-coronavirus-has-spread/.

[9] Canadian Pandemic Influenza Preparedness Task Group (2018). Canadian Pandemic Influenza Preparedness Planning Guidance for the Health Sector. Government of Canada. https://www.canada.ca/content/dam/phac-aspc/migration/phac-aspc/cpip-pclcpi/assets/pdf/report-rapport-02-2018-eng.pdf.

[10] National Institute for Public Health and the Environment (RIVM) (2020). Current information about COVID-19 (novel coronavirus). https://www.rivm.nl/en/novel-coronavirus-covid-19/current-information.

[11] Dutch College of General Practitioners (2020). [Reliable information about illness and health] *Betrouwbare informatie over ziekte en gezondheid* (in Dutch). https://www.thuisarts.nl/corona.

[12] Schers H, van Weel C, Van Boven K (2020). The COVID-19 pandemic in the Netherlands: impact on primary care. https://deepblue.lib.umich.edu/bitstream/handle/2027.42/154735/Schers%20Deep%20Blue%20article%20file.pdf?sequence=1&isAllowed=y.

[13] Pandemic Influenza Preparedness Team, Department of Health (2011). Scientific Summary of Pandemic Influenza & its Mitigation. https://assets.publishing.service.gov.uk/government/uploads/system/uploads/attachment_data/file/215666/dh_125333.pdf.

[14] US Department of Health and Human Services (2017). Pandemic Influenza Plan 2017 Update. https://www.cdc.gov/flu/pandemic-resources/pdf/pan-flu-report-2017v2.pdf.

[15] Kamerow D (2020). Covid-19: Don't forget the impact on US family physicians. BMJ.

[16] United States Census Bureau (2019). Health Insurance Coverage in the United States: 2018. https://www.census.gov/library/publications/2019/demo/p60-267.html.

[17] Cutler D (2020). How will COVID-19 affect the health care economy?. JAMA.

[18] The Green Center (2020). Quick Covid-19 survey: clinician survey. https://www.green-center.org/covid-survey.

[19] College of Family Physicians of Canada (2020). Family physicians’ response to the COVID-19 pandemic. May 2020. https://portal.cfpc.ca/ResourcesDocs/uploadedFiles/Research/Covid-19-Member-Survey-ENG-Final.pdf.

[20] McNamara A (15 Apr 2020). Why are non-COVID related deaths on the rise? Science Focus [online]. https://www.sciencefocus.com/news/why-are-non-covid-related-deaths-on-the-rise/.

[21] Pratt E (5 May 2020). Excess deaths: people who are dying because of COVID-19 — but not from it. Healthline [online]. https://www.healthline.com/health-news/excess-deaths-from-covid19-pandemic.

[22] Giles C (22 Apr 2020). UK coronavirus deaths more than double official figure, according to FT study. Financial Times [online]. https://www.ft.com/content/67e6a4ee-3d05-43bc-ba03-e239799fa6ab.

[23] The Netherlands Healthcare Authority (2020). [Analysis of the consequences of the corona crisis for regular healthcare] *Analyse van de gevolgen van de coronacrisis voor de reguliere zorg* (in Dutch). https://puc.overheid.nl/nza/doc/PUC_306627_22/1/.

[24] Griffin S (2020). Covid-19: "Staggering number" of extra deaths in community is not explained by covid-19. BMJ.

[25] Petterson S, Westfall JM, Miller BF (2020). Projected deaths of despair during the coronavirus recession. Well Being Trust. Robert Graham Center. https://wellbeingtrust.org/wp-content/uploads/2020/05/WBT_Deaths-of-Despair_COVID-19-FINAL-FINAL.pdf.

[26] Garcia S, Albaghdadi MS, Meraj PM (2020). Reduction in ST-segment elevation cardiac catheterization laboratory activations in the United States during COVID-19 pandemic. J Am Coll Cardiol.

[27] De Filippo O, D'Ascenzo F, Angelini F (2020). Reduced rate of hospital admissions for ACS during Covid-19 outbreak in northern Italy. N Engl J Med.

[28] McDonald HI, Tessier E, White JM (2020). Early impact of the coronavirus disease (COVID-19) pandemic and physical distancing measures on routine childhood vaccinations in England, January to April 2020. Euro Surveill.

[29] Bramer CA, Kimmins LM, Swanson R (2020). Decline in child vaccination coverage during the COVID-19 pandemic - Michigan Care Improvement Registry, May 2016-May 2020. MMWR Morb Mortal Wkly Rep.

[30] Westfall JM, Strange K, DeVoe J (7 Apr 2020). Coronavirus: Family physicians provide telehealth care at risk of bankruptcy. USA Today [online]. https://www.usatoday.com/story/opinion/2020/04/07/coronavirus-family-physicians-provide-telehealth-care-risk-bankruptcy-column/2942535001/.

[31] Kamerow D (2020). Covid-19: Don't forget the impact on US family physicians. BMJ.

[32] Ontario Medical Association (2020). Up to half of Ontario’s doctors responding to survey may have to close their offices. https://www.oma.org/section/search/practice-impact-survey?type=news_items.

[33] Boothby L (14 March 2020). COVID-19 could bankrupt clinics because of low fees for virtual visits, Edmonton doctors’ group says. Edmonton Journal [online]. https://edmontonjournal.com/news/politics/covid-19-edmonton-doctors-group-says-virtual-visits-could-close-clinics-because-of-low-fees.

[34] Durski KN, Osterholm M, Majumdar SS (2020). Shifting the paradigm: using disease outbreaks to build resilient health systems. BMJ Glob Health.

[35] World Health Organization (1978). Declaration of Alma-Ata. International Conference on Primary Health Care. Alma-Ata, USSR: WHO. https://www.who.int/publications/almaata_declaration_en.pdf?ua=1.

[36] World Health Organization (2018). Astana Declaration: from Alma-Ata towards universal health coverage and the Sustainable Development Goals.

[37] Government of Canada (2020). Flu (influenza): FluWatch surveillance. https://www.canada.ca/en/public-health/services/diseases/flu-influenza/influenza-surveillance.html.

[38] ESR (2020). SHIVERS influenza (flu) and respiratory virus research. https://www.esr.cri.nz/our-research/research-projects/shivers-project/.

[39] Netherlands Institute for Health Services Research (2020). NIVEL Primary Care Database - Sentinel Practices 2013. https://nivel.nl/sites/default/files/bestanden/Peilstations-jaarverslag-2013-Engels.pdf.

[40] RCGP (2020). Research and Surveillance Centre. https://www.rcgp.org.uk/clinical-and-research/our-programmes/research-and-surveillance-centre.aspx.

[41] United States Centers for Disease Control and Prevention (2020). Flu activity and surveillance. https://www.cdc.gov/flu/weekly/fluactivitysurv.htm.

[42] Nuffield Department of Primary Care Sciences (2020). Coronavirus analysis and resources. https://www.phc.ox.ac.uk/covid-19.

[43] Australian Sentinel Practices Research Network (2020). Australian Sentinel Practices Research Network: About. https://aspren.dmac.adelaide.edu.au.

